# Present-day oxidative subsidence of organic soils and mitigation in the Sacramento-San Joaquin Delta, California, USA

**DOI:** 10.1007/s10040-016-1391-1

**Published:** 2016-03-28

**Authors:** Steven J. Deverel, Timothy Ingrum, David Leighton

**Affiliations:** HydroFocus, Inc., 2827 Spafford Street, Davis, CA 95618 USA

**Keywords:** Subsidence, Geohazard, USA, Land use, Soil processes

## Abstract

Subsidence of organic soils in the Sacramento-San Joaquin Delta threatens sustainability of the California (USA) water supply system and agriculture. Land-surface elevation data were collected to assess present-day subsidence rates and evaluate rice as a land use for subsidence mitigation. To depict Delta-wide present-day rates of subsidence, the previously developed SUBCALC model was refined and calibrated using recent data for CO_2_ emissions and land-surface elevation changes measured at extensometers. Land-surface elevation change data were evaluated relative to indirect estimates of subsidence and accretion using carbon and nitrogen flux data for rice cultivation. Extensometer and leveling data demonstrate seasonal variations in land-surface elevations associated with groundwater-level fluctuations and inelastic subsidence rates of 0.5–0.8 cm yr^–1^. Calibration of the SUBCALC model indicated accuracy of ±0.10 cm yr^–1^ where depth to groundwater, soil organic matter content and temperature are known. Regional estimates of subsidence range from <0.3 to >1.8 cm yr^–1^. The primary uncertainty is the distribution of soil organic matter content which results in spatial averaging in the mapping of subsidence rates. Analysis of leveling and extensometer data in rice fields resulted in an estimated accretion rate of 0.02–0.8 cm yr^–1^. These values generally agreed with indirect estimates based on carbon fluxes and nitrogen mineralization, thus preliminarily demonstrating that rice will stop or greatly reduce subsidence. Areas below elevations of –2 m are candidate areas for implementation of mitigation measures such as rice because there is active subsidence occurring at rates greater than 0.4 cm yr^–1^.

## Résumé

L’affaissement des sols organiques dans le Delta de Sacramento-San Joaquin menace la durabilité du système d’approvisionnement en eau et de l’agriculture de la Californie (Etats-Unis d’Amérique). Des données d’altitude de la surface topographique ont été collectées dans le but d’estimer le taux d’affaissement actuel des sols et d’évaluer l’occupation des sols par des rizières en tant que moyen d’atténuation de l’affaissement des sols. Pour décrire les taux actuels d’affaissement des sols à l’échelle du Delta, le modèle SUBCALC développé précédemment a été affiné et calé grâce à l’utilisation des données récentes sur les émissions de CO_2_ et des variations d’altitude de la surface topographique enregistrées par des extensomètres. Les données de variations d’altitude de la surface topographique ont été évaluées par rapport aux estimations indirectes de l’affaissement du sol et de son accroissement basées sur les données de flux de carbone et d’azote au niveau des rizières. Les données d’extensiométrie et de nivellement montrent que les variations saisonnières de l’altitude de la surface topographique associées aux fluctuations des niveaux piézométriques et des taux d’affaissement inélastique du sol compris entre 0.5 et 0.8 cm an^–1^. Le calage du modèle SUBCALC indique une précision de ±0.10 cm an ^–1^ là où la profondeur de la nappe, la teneur en matière organique du sol et la température sont connues. Les estimations régionales de l’affaissement du sol sont compris entre <0.3 à >1.8 cm an^–1^. L’incertitude principale est la distribution de la teneur en matière organique du sol qui conduit à un lissage spatial dans la cartographie des taux d’affaissement des sols. L’analyse des données de nivellement et d’extensiométrie dans les rizières conduit à un taux d’accroissement estimé entre 0.02 et 0.8 cm an^–1^. Ces valeurs sont généralement en accord avec les estimations indirectes basées sur les flux de carbone et de la minéralisation de l’azote qui indiquent a priori que le riz stoppera ou réduira de manière substantielle l’affaissement du sol. Les zones d’altitude inférieure à –2 m sont des zones candidates pour la mise en œuvre de mesures d’atténuation telles que la culture du riz car il s’y produit un affaissement du sol actif avec des taux supérieurs à 0.4 cm an^–1^.

## Resumen

La subsidencia de los suelos orgánicos en el Delta de Sacramento-San Joaquín amenaza la sostenibilidad del sistema de suministro de agua y la agricultura en California (EE.UU.). Se recolectaron datos de elevación de la superficie del terreno para evaluar las tasas de subsidencia actuales y evaluar el cultivo de arroz como un uso de la tierra para la mitigación de la subsidencia. Se refinó y calibró el modelo SUBCALC desarrollado previamente para describir las tasas de la subsidencia en el ancho actual del Delta, usando los datos más recientes de las emisiones de CO_2_ y los cambios en la elevación de la superficie del terreno medidos con extensómetros. Se evaluaron los datos de cambio de la elevación de la superficie del terreno con respecto a las estimaciones indirectas de la subsidencia y la acumulación de carbono a partir de datos y el flujo de nitrógeno para el cultivo de arroz. Los datos de extensómetros y de nivelación demuestran variaciones estacionales en las elevaciones de la superficie terrestre asociados a las fluctuaciones del nivel del agua subterránea y tasas de subsidencia inelásticas de 0.5 a 0.8 cm año^–1^. La calibración del modelo SUBCALC indicó una precisión de ±0.10 cm año^–1^, donde son conocidas la profundidad del agua subterránea, el contenido de materia orgánica del suelo y la temperatura. Las estimaciones regionales de subsidencia varía de <0.3 a >1.8 cm yr^–1^. La principal incertidumbre es la distribución del contenido de materia orgánica en el suelo que resulta del promedio espacial en el mapeo de las tasas de subsidencia. El análisis de los datos de nivelación y extensómetro en los campos de arroz resultó en una tasa de acreción estimada entre 0.02 y 0.8 cm yr^–1^. Estos valores en general concuerdan con las estimaciones indirectas basadas en los flujos de carbono y nitrógeno, lo que demuestra en forma preliminar que la mineralización del arroz va a detener o reducir en gran medida la subsidencia. Las áreas por debajo de elevaciones de –2 m son áreas examinadas para la implementación de medidas de mitigación, tales como el cultivo de arroz debido a que no hay lugar para un ritmo de subsidencia activa de más de 0.4 cm año^–1^.

## 摘要

沙加缅度河—圣华金河三角洲有机土壤的下沉威胁着(美国)加利佛尼亚州供水系统和农业的可持续性。收集了地面高程资料以评价目前的下沉速率并且评估了土地种植水稻减缓下沉的作用。为了描述三角洲范围内目前的下沉速度,采用目前的CO_2_排放资料和伸长仪测量的地面高程变化对先前开发的SUBCALC模型进行了改进和校正。针对利用水稻种植碳和氮通量资料得到的间接下沉和吸积量估算结果,对地表高程变化资料进行了评估。伸长仪和水准测量资料显示了地表高程变化的季节性变化与地下水位波动及0.5 至 0.8 cm yr^–1^的非弹性下沉速度有关。在地下水深度、土壤有机物含量和温度已知的情况下,SUBCALC模型校正表明精确度为±0.10 cm yr^–1^。区域下沉的估算范围为 < 0.3 至 >1.8 cm yr^–1^。主要的不确定性为土壤有机物含量的分布状况,这种不确定性会导致绘制下沉速度时的空间平均值。水稻田中的水准测量和伸长仪资料分析得出的吸积率估算值为0.02 至 0.8 cm yr^–1^。这些值通常与根据碳通量和氮矿化作用得到的间接估算值一致,从而初步展示,水稻能阻止或减少下沉。高程低于–2 m的区域是实施减缓措施诸如种植水稻的候选区域,因为在这些区域将会有速度大于0.4 cm yr^–1^的下沉。

## Resumo

A subsidência dos organossolos no Delta dos rios Sacramento-San Joaquin põe em risco a sustentabilidade do sistema de abastecimento de água e da agricultura do estado da Califórnia (EUA). Foram coletados dados de elevação da superfície do solo para estimar as taxas atuais de subsidência e avaliar o uso de cultivo de arroz para mitigar a subsidência. Para simular as taxas atuais de subsidência da região do Delta, o modelo SUBCALC, desenvolvido anteriormente, foi refinado e calibrado utilizando dados recentes de emissões de CO_2_ e mudanças na elevação do terreno obtidas com extensômetros. Os dados de mudança na elevação foram avaliados em relação a estimativas indiretas de subsidência e acreção, utilizando dados de fluxo de carbono e nitrogênio no cultivo de arroz. Os dados dos extensômetros e de nivelamento demonstraram variações sazonais de elevações do terreno, associados a flutuações do nível da água subterrânea e taxas de subsidência inelásticas que variam de 0.5 a 0.8 cm ano^–1^. A calibração do modelo SUBCALC indicou precisão de ±0.10 cm por ano^–1^, onde a profundidade das águas subterrâneas e o teor de matéria orgânica e temperatura do solo eram conhecidos. As estimativas regionais de subsidência variam de <0.3 a >1.8 cm ano^–1^. A principal incerteza nas estimativas de subsidência modeladas é a distribuição do conteúdo de matéria orgânica do solo. Porem, as taxas mapeadas de subsidência representam médias em áreas de solos mapeados com teor de materia organica semelhantes. Análises dos dados de nivelamento e dos extensômetros em campos de produção de arroz, resultaram em uma taxa de acreção estimada de 0.02 a 0.8 cm por ano^–1^. Estes valores geralmente concordam com estimativas indiretas baseadas em fluxos de carbono e a mineralização do nitrogênio, portanto, indicando que o cultivo de arroz ira parar ou reduzira significativamente o processo de subsidência. Áreas com elevação inferior a –2 m são candidatas para a implementação de medidas de mitigação como o cultivo de arroz, por haver subsidência ativa ocorrendo com taxas superiores a 0.4 cm por ano^–1^.

## Introduction

Worldwide, many deltas are sinking due to reduced aggradation and compaction and soil loss resulting from fluid withdrawal, drainage, and oxidation of organic matter (Syvitski et al. 2009). Drainage of organic soils has resulted in soil subsidence due to changes in physical conditions and enhanced rates of microbial decomposition (Hirano et al. 2012; Rojstaczer and Deverel 1993; Stephens et al. 1984). In total, 14–20 % of the world’s organic soils or peatlands are currently drained for agriculture or forestry (Strack 2008).

Organic soils or histosols are defined by the Food and Agriculture Organization as having 40 cm or more of organic materials with an organic carbon content of 12–18 % or more. The terms organic soil and peat are often used interchangeably and generally refer to a soil which formed under saturated wetland conditions and is acidic and rich in humus. Peat soils cover an estimated 400 million ha, equivalent to 3 % of the Earth’s land surface (Kaat and Joosten 2009), with most peatlands occurring in the northern hemisphere.

Farming and subsidence of peats has been studied in multiple locations. The oldest records are from the Netherlands where peat soils were drained starting between the 9th and 14th centuries. Schorthorst (1977) documented subsidence rates varying from 0.17 to 0.7 cm yr^–1^ since the 1800s in these peat soils. Stephens et al. (1984) summarized worldwide subsidence rates which ranged from less than 0.5 to 10 cm yr^–1^ in California, Louisiana, Michigan, New York, Indiana and Florida in the USA and the Netherlands, Republic of Ireland, Norway, England (UK), Israel and Russia. Subsidence in agricultural peatlands has also been studied and estimated in New Zealand (Schipper and McLeod 2002), Southeast Asia (e.g. Hooijer et al. 2012) and Italy (Zanello et al. 2011; Gambolati et al. 2006).

Reported causes of peat subsidence include (1) shrinkage due to dewatering, (2) consolidation due to loss of buoyant force and loading, (3) wind and water erosion, (4) oxidation of soil organic matter and (5) burning. Ewing and Vepraskas (2006) differentiated between relatively larger rates of primary subsidence or shrinkage upon drainage and lower rates of secondary subsidence or oxidation. During the 1970s, Schorthorst (1977) reported that compaction, shrinkage, and microbial oxidation caused 28, 20, and 52 % of subsidence in the Netherlands, respectively.

US Department of Agriculture and University of Florida researchers extensively studied subsidence in Florida Everglades peats (Stephens et al. 1984) and reported that oxidation accounted for 53 % of historical subsidence. Florida researchers also demonstrated the relation of subsidence and carbon dioxide production (Stephens and Stewart 1976), soil temperature and moisture (Knipling et al. 1970; Volk 1973) and microbial activity (Tate 1979, 1980a, 1980b). Stephens et al. (1984) and Couwenberg and Hooijer (2013) reported an inverse correlation of subsidence rates, and oxidative carbon loss, to depth to groundwater.

### Sacramento-San Joaquin Delta

**Fig. 1:**
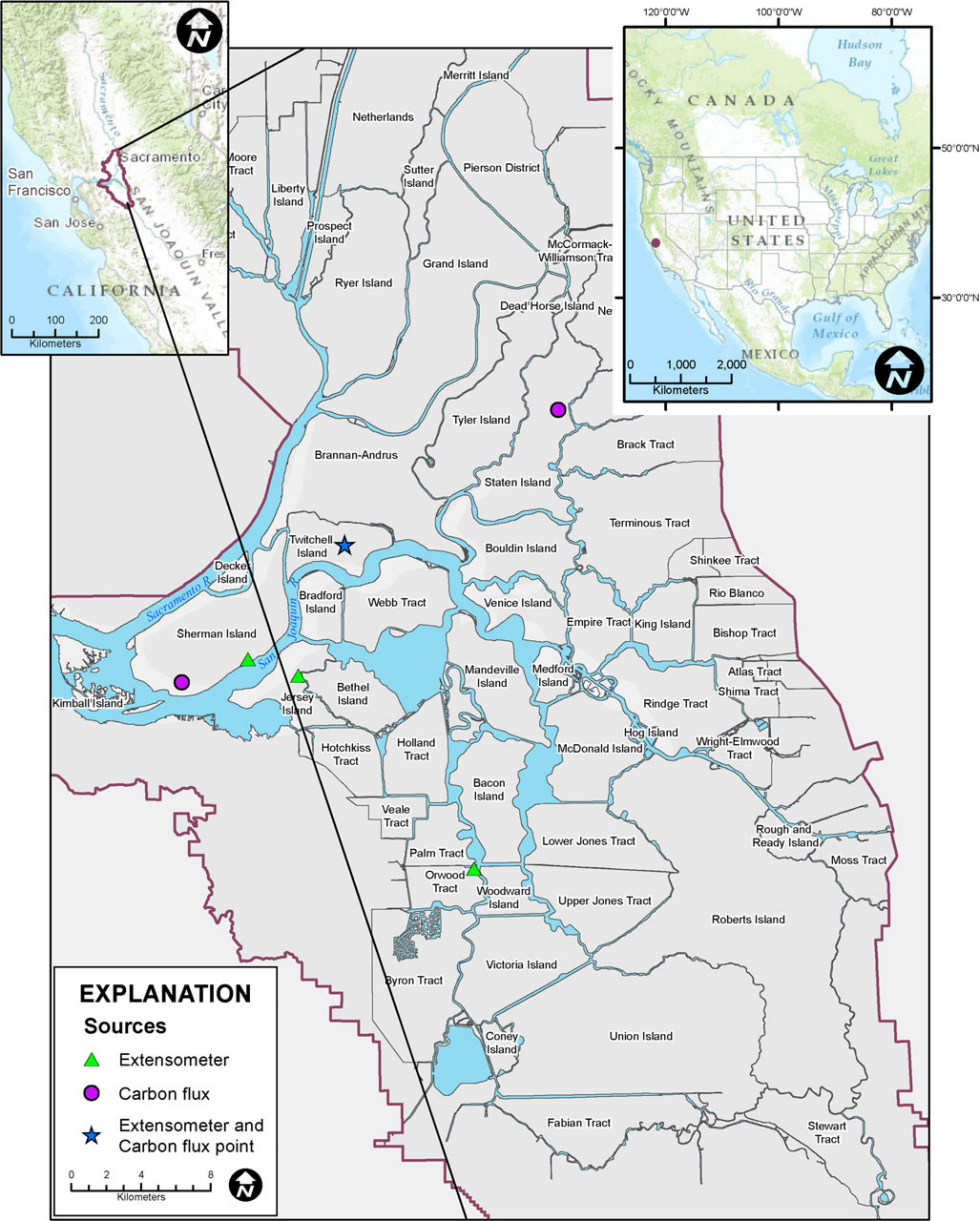
Map showing locations of the Sacramento-San Joaquin Delta and subsidence and carbon fluxes measurements

**Fig. 2:**
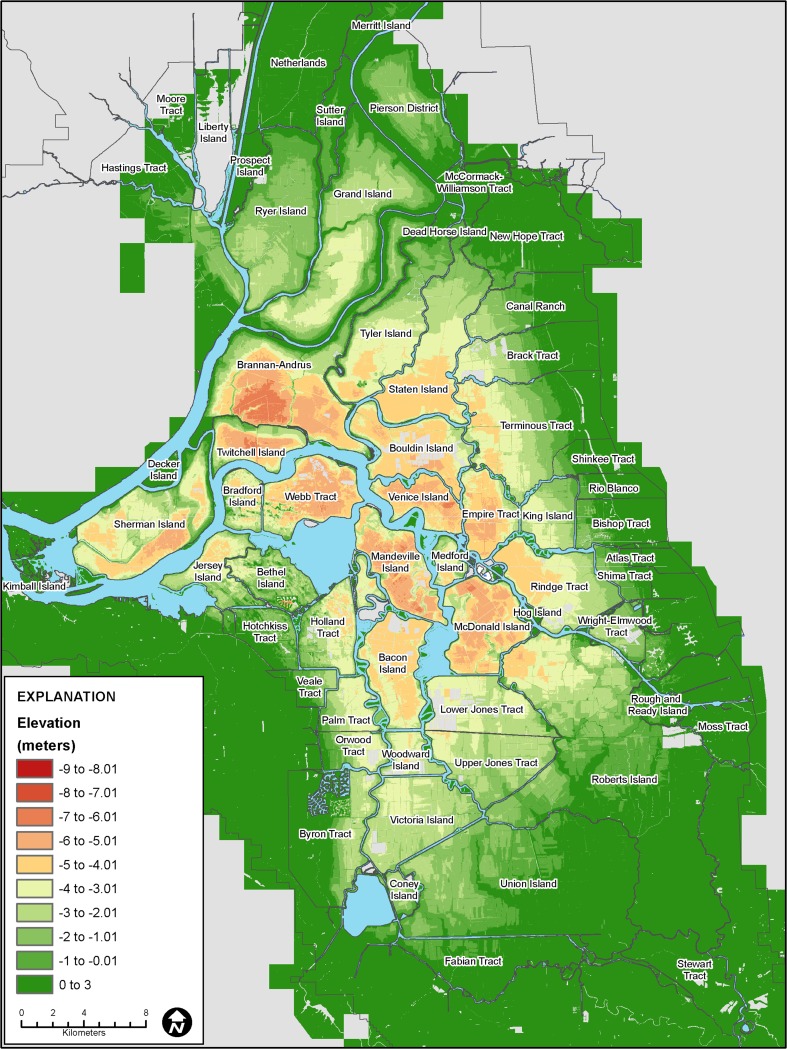
Map showing land surface elevations where organic soils predominated at the time of data collection in the Sacramento-San Joaquin Delta (based on LiDAR data from CDWR 2007)

Substantial understanding and quantification of subsidence has occurred since the early 1900s in the Sacramento-San Joaquin Delta, California, USA (hereafter Delta; Fig. 1). Subsidence of organic and highly organic mineral soils (hereafter referred to as organic soils or peat) is a primary landscape-altering process that threatens Delta infrastructure and water supply for over 25 million Californians. Drainage and cultivation of Delta soils since 1850 resulted in subsidence on over 60 islands from 1 to over 8 m (Thompson 1957; Deverel and Leighton 2010; Fig. 2). Key factors influencing subsidence include percent soil organic matter, depth of peat, year of initial drainage and management practices such as burning or growing crops that leave the soil exposed to wind erosion (Deverel and Leighton 2010).

The overall objectives of this paper are to: (1) summarize the state of the science for oxidative subsidence of Delta peat soils and report the analysis of recent land-surface elevation data, refinement and recalibration of the subsidence model SUBCALC, (2) estimate present-day subsidence rates and (3) preliminarily assess rice cultivation for stopping or reducing subsidence.

Delta peat soils formed from decaying wetland plants (Atwater 1982; Shlemon and Begg 1975; Drexler et al. 2009a). During the 6,000–7,000 years prior to the 1850s, about 5 billion m^3^ of tidal marsh sediment accumulated in the Delta (Deverel and Leighton 2010; Mount and Twiss 2005). Since the mid-19th century, half of this volume disappeared (Deverel and Leighton 2010; Mount and Twiss 2005). Present-day soils reflect organic matter accumulation through millennia, spatially variable fluvial deposition and oxidation; thus, soil type and organic matter content vary substantially (Deverel and Leighton 2010). Highly organic mineral surface soils generally predominate in the western and northern Delta and true surface organic soils, or histosols predominate in the central, eastern and southern-central Delta. The lowest organic matter content soils, which subside at relatively low rates, generally predominate in areas drained prior to 1880 near the Sacramento River where there was greater fluvial deposition (Deverel and Leighton 2010). Higher subsidence rates are associated with more recently drained and higher-organic matter soils in the central Delta where there was less fluvial deposition (Deverel and Leighton 2010).

Using data presented in Atwater (1982) and boring logs, Deverel and Leighton (2010) and Deverel et al. (2015) presented maps of peat thickness. The thickest peat resides in the western and northwestern Delta where thicknesses range to over 7 m on Sherman Island (Fig. 1). Three to over 7 m of peat remains on Ryer, southern Grand, western Brannan, and Twitchell islands (Fig. 1). For most of the central, eastern and southern-central Delta, less than 1–2 m of peat remains (see Fig. 4 in Deverel et al. 2015).

Deverel and Rojstaczer (1996) reported that microbial oxidation of organic matter is the primary present-day cause of subsidence. Consistently, Rojstaczer and Deverel (1995) and Deverel and Leighton (2010) demonstrated that spatial variations in soil organic matter content ranged from 4 to 60 % and explained over 55 % of the variation in average subsidence rates from 1978 to 2006. Deverel and Leighton (2010) assessed recent and historic causes of and factors affecting subsidence rates using elevation and soils data collected during 2006 and data reported in Rojstaczer et al. (1991), Rojstaczer and Deverel (1995), Deverel and Rojstaczer (1996) and the University of California (Weir 1950). Deverel and Leighton (2010) developed a computer model, SUBCALC, and geographic information system (GIS) to simulate Delta-wide subsidence. The SUBCALC model was calibrated using historic land-surface elevation data collected on three islands in the central Delta (Bacon and Mildred islands and Jones Tract) by Weir (1950) and Sherman Island in the western Delta (Rojstaczer et al. 1991).

Subsidence rates have decreased with time associated with decreasing soil organic matter content and changing land-management practices (Deverel and Leighton 2010). Prior to the early 1960s, burning and wind erosion caused soil loss. Burning no longer occurs, and there is minimal wind erosion. Wind erosion was associated with high-velocity spring winds and bare asparagus fields (Schultz and Carlton 1959; Schultz et al. 1963; Carlton and Schultz 1966). Asparagus was widely planted in the Delta during the 1920s through the 1950s. Due to economic reasons, since the 1960s, asparagus cultivation decreased to a small area. Model results and their agreement with measurements demonstrate that oxidation accounts for the majority of recent subsidence and the remaining portion is due to consolidation (Deverel and Leighton 2010). The application of Michaelis–Menton kinetics was used to simulate oxidation of soil organic carbon. Consistently, Volk (1973) reported that Michaelis–Menton kinetics appropriately described oxidation of peat soils in the Florida Everglades.

Little has been documented about consolidation, a secondary cause of organic soil subsidence. Water in organic soils is held in three phases: (1) intercellular, (2) inter-particle water in micropores, and (3) bound or absorbed. Consolidation expulses pore water and particles rearrange (Hobbs 1986). As farmers deepened drainage ditches to compensate for land-surface elevation loss due to oxidation, wind erosion and burning, organic soils consolidated due to dewatering resulting from increased drainage-ditch depth, which reduces pore pressure and buoyancy, thus transferring load to the soil skeleton. Drexler et al. (2009b) presented evidence for consolidation below the upper oxidized layer on farmed subsided islands.

To estimate consolidation of subsurface deposits, Deverel and Leighton (2010) assumed compaction proceeds similar to dewatering and irreversible consolidation of a vertical soil column as described by Terzaghi (1925). They employed Terzaghi’s effective stress principle and extensometer data on Twitchell Island to estimate consolidation in SUBCALC using a linear model relating compaction to the change in hydraulic head. The estimated percentages of the different causes of subsidence came from model simulations of elevation change data from the 1920s to 2006.

Since the late 1990s, researchers have investigated large-scale and small-scale spatial and temporal trends of subsidence on Delta islands and levees using remotely sensed data. Attempts to use satellite radar data for investigating oxidative subsidence of organic soils suffered from rapid decorrelation in agricultural areas (Cohen et al. 1998; Brooks et al. 2012)—for example, Brooks et al. (2012) showed limited ability to estimate subsidence on Delta island interiors (see Fig. 2 in their paper) presumably due to decorrelation. The Brooks et al. (2012) estimates were primarily for Delta levees around the periphery of islands where decorrelation has less of an effect. The applicability of satellite InSar is primarily limited to more stable structures such as levees.

More recently, the relatively longer L-band (23.8 cm) wavelength of the UAVSAR, combined with regular acquisitions, high spatial resolution and data processing techniques developed for low coherence regions, show promise for application of radar interferometry to monitor subsidence in Delta agricultural lands on island interiors at sub-centimeter vertical resolution levels on organic soils (Jones et al. 2011, 2012). UAVSAR data have been used to estimate rates of elevation change on Sherman Island organic soils (Priyanka Sharma, Jet Propulsion Laboratory, unpublished data, 2015). Values ranged from 0 to 5 cm yr^–1^ and the spatially averaged rate was 1.5 cm yr^–1^. The UAVSAR-estimated rates were generally consistent with ground-based estimates described here and Deverel and Leighton (2010) and Rojstaczer and Deverel (1995). Terrestrial light detection and ranging technology (Terrestrial LiDAR) has also been used in the Delta to quantify short-term local-scale levee deformation (e.g. Bawden et al. 2014).

#### Effects of subsidence and mitigation

By reducing the landmass and resistance to hydraulic pressure from adjacent channels, subsidence has contributed to levee failure and island inundation. Weir (1950) reported the results of land surveys and field observations on three central Delta islands from 1922 to 1948 where elevations declined 1.8–2.3 m, resulting in elevations ranging from 3 to 3.4 m below sea level. Weir (1950) warned about increased seepage, levee instability and island flooding associated with subsidence. At the time of Weir’s publication, 50 islands had flooded primarily due to overtopping of levees. From 1930 to the early 1980s, over 50 Delta islands or tracts flooded—the majority due to levee foundation instability (Prokopovitch 1985)—whereas from 1900 to 2006, over 100 island levees failed (Gaddie et al. 2006; Florsheim and Dettinger 2007), causing local infrastructural damage which historically cost hundreds of millions of dollars (Prokopovitch 1985).

The flooding of the 4,860-ha Jones Tract due to a levee breach that occurred during the early morning of June 2004 illustrates recent consequences. About $90 million were expended by the State of California to close the levee breach and complete removal of 197 million m^3^ of flood water by December 2004. Before island reclamation was complete, litigation began when BNSF Railway alleged that the State of California’s operation of the State Water Project resulted in channel scour that induced levee failure. Jones Tract landowners and others joined in the lawsuit. In its statement of decision and judgement, the State Superior Court ruled against the plaintiffs, dismissed the theory of State-Water-Project channel scour and stated that: “Subsidence resulting from the loss of peat soil over the years makes Delta islands more susceptible to flooding due to levee failure”.

Island flooding in the western Delta may also cause eastward movement of saline water into the Delta, and thus impede water exports—for example, levee failure and flooding on Brannan-Andrus Island (Fig. 1) in June 1972 caused movement of salt water into the Delta (Cook and Coleman 1973), resulting in cessation of state and federal water exports. An additional 370 million m^3^ of water was released from reservoirs to mitigate the salinity intrusion. The total cost of flooding was over $97 million in 2015 dollars.

Delta subsidence will continue until management practices are adopted that stop subsidence or the organic deposits disappear. Continuing subsidence can make farming more difficult and expensive. For example, as peat disappears, drainage ditches may be excavated into underlying mineral sediments which can be unstable. Also, greater seepage due to increased hydraulic gradients and flow under levees is resulting in larger marginally or non-farmable acreage (Deverel et al. 2015).

Hydraulic forces on and seepage through and under levees will increase with continuing subsidence and sea level rise (Deverel et al. 2007a, 2014, 2015). Increased seepage under levees onto islands will decrease levee stability. Deverel et al. (2007b) predicted that seepage onto Twitchell Island (Fig. 1) will increase by 22–34 % during the next several decades. Levee failure or instability from seepage occurs when hydraulic gradients are large enough to erode or move levee internal and/or foundational materials. The Delta Risk Management Strategy (CDWR 2009) estimated the combined probability of levee failure and island flooding from earthquake, high-water flooding, and sunny-day levee failure for most of the deeply subsided central and western Delta ranges from 53 % to over 84 % during the next 20 years. Economic costs of future Delta levee failures were estimated in billions of dollars.

Island drainage volumes and dissolved organic carbon loads will increase with continuing subsidence (Deverel et al. 2007a) and drainage costs will increase due to greater pumping lifts and volumes. Oxidation of organic soils generates dissolved organic carbon which forms, upon treatment for drinking, carcinogenic disinfection byproducts (Fleck et al. 2004; Deverel et al. 2007b). Winter and spring rains and irrigation mobilize dissolved organic carbon and trihalomethane precursor loads into island drainage water (Deverel et al. 2007b). Ongoing oxidative subsidence therefore perpetuates the annual cycle of generation of dissolved organic carbon in soil and mobilization to drainage water which is exported to Delta channels which deliver drinking water to 25 million Californians via the State Water Project (CDWR 2015). Similar processes apparently operate to generate methyl mercury, and data presented in Heim et al. (2009) indicate that methyl mercury loads from Delta islands will increase with continuing subsidence.

Managed and permanently flooded wetlands will stop and reverse the effects of subsidence (Deverel et al. 1998, 2014; Miller et al. 2000, 2008). These wetlands accumulate carbon, and accretion rates are about 3 cm yr^–1^ (Deverel et al. 2014); wetlands near levees will reduce seepage onto islands (Deverel et al. 2014).

Agriculture is the predominant land use in the Delta and is important to the local economy. However, current farming practices which require an aerated root zone cause subsidence by exposing organic soils to oxygen; therefore, agricultural practices that stop subsidence are highly desirable. The original impetus for investigating rice as a subsidence mitigation land use came from Miller et al. (2000) who indicated that wetlands which were flooded from early spring through midsummer resulted in no net carbon loss. Rice growers use a similar water management practice, flooding rice fields during the warmest months when soil oxidation rates are highest. In the past, cool night temperatures precluded Delta rice cultivation; however, development of cold-tolerant rice varieties resulted in increased Delta rice production with yields generally comparable to other rice-growing areas in California. Rice has been successfully grown on about 3,000 acres on central and eastern Delta islands since the mid-1990s.

Heightened recent interest in subsidence mitigation prompted further investigation into rice production on state-owned Twitchell Island in 2009. Micrometeorological data presented by Hatala et al. (2012) and soil nitrogen dynamics reported by Kirk et al. (2015) suggest that rice cultivation will greatly reduce oxidative subsidence in Delta organic soils. Extensometer and leveling data collected in rice fields and an adjacent cornfield are reported here.

#### Need for dissemination of quality information

Misinformation about present-day Delta subsidence points to the need for collection and dissemination of high quality information. For example, in the Economic Sustainability Plan, the Delta Protection Commission (2012) cited work which attempted to delineate areas of active subsidence based on comparison of 2007 LiDAR data (CDWR 2007) and USGS Quadrangle maps surveyed between 1974 and 1976 (California Central Valley Flood Control Association 2011). The accuracy of the 2007 LiDAR data is about ±0.15 m. The error due to estimating the elevations from the 1974 and 1976 quadrangle contour maps is about one-half of the contour interval (1.5 m) for the topographic maps or 0.76 m (J. Vukovitch, USGS, Denver, personal communication, 1996). During 1974 to 2007, subsidence rates ranged from about 1–3 cm per yr^–1^, resulting in 0.3–0.9 m of elevation change which is similar to the estimation error from the Quadrangle maps and LiDAR data.

Since publication of Deverel and Leighton (2010), additional land-surface elevation-change data has been collected on selected Delta islands; also, greenhouse gas emissions have been measured. While there is a need to assess, analyze and synthesize these data and improve subsidence modeling capability, the overall approach taken here was to collect and analyze land-surface elevation data in rice, corn and pasture fields, refine and recalibrate SUBCALC and estimate present-day Delta subsidence rates.

## Data sources and methods

### Land-surface elevation data

#### Extensometer data

To monitor small-scale variations in land surface elevation during 2009–2015, HydroFocus personnel installed and operated two extensometers on Twitchell Island, one in a rice field and one in the cornfield adjacent to the rice field (Fig. 1). The cornfield was converted to a wetland in 2014. At the extensometer location on Sherman Island (Fig. 1) described in Deverel and Rojstaczer (1996), new instrumentation recorded elevation changes starting in 2011. At all locations, land-surface elevations were measured relative to the extensometer structure which was anchored below the peat.

In the rice field, a steel base support pipe was driven to refusal into the mineral layer underlying the peat soil. A modified sedimentation-erosion table (SET; Boumans and Day 1993) was inserted into a grooved stainless steel sleeve in the base support pipe which ensured instrument stability and replacement to the exact same position after movement to accommodate field operations. The SET arm extended horizontally about 1 m and was adjusted to level. A metal rod with a 5-inch-diameter (12.7 cm) metal disk that rested on the ground freely moved vertically in a sleeve on a metal plate at the end of the arm. HydroFocus personnel fastened a Macro Sensors GHSI 750 linear variable differential transformer (LVDT) to the rod above the plate. The piston arm rested on the plate so that the sensor body would move with the rod and the piston arm would remain stationary. A Campbell CR510 data logger recorded LVDT measurements every 15 min. In the cornfield, HydroFocus personnel constructed an extensometer similar to the one described in Deverel and Rojstaczer (1996).

#### Leveling data

Annual leveling surveys were conducted at seven locations in the Twitchell Island rice fields during 2009–2013 (Fig. 1) by California Department of Water Resources personnel. Spirit leveling surveys in multiple directions relative to fixed monuments anchored in the mineral material were conducted in the spring of each year after cultivation and before flooding. Land-surface elevations were determined using GPS in 2001 and 2012 at seven monitoring-well locations on Twitchell Island (Fig. 1).

### Modeling of delta subsidence

Deverel and Leighton (2010) developed the computer model, SUBCALC, to integrate available data and quantify and simulate subsidence rates and causes. SUBCALC simulates aerobic microbial oxidation of organic carbon, consolidation, wind erosion and burning. Present-day subsidence is the result of oxidation and consolidation. SUBCALC simulates microbial oxidation of soil organic carbon to carbon dioxide using Michaelis–Menton (M–M) enzyme kinetics in which the rate of soil organic-matter oxidation is limited by soil organic carbon content (Browder and Volk 1978):

1 $$ \frac{V}{V_{\max }}=\frac{\left[S\right]}{K_{\mathrm{m}}+\left[S\right]} $$

Michaelis-Menton equation (Eq. 1) parameters, *K*_m_ (the M–M constant) and *V*_max_, (the maximum oxidation rate), and effects of temperature were originally estimated based on data reported in Deverel and Rojstaczer (1996). The [*S*] term (substrate concentration term) is the soil organic carbon fraction. For each annual time step, the different contributions to subsidence (e.g. oxidation and consolidation) were estimated based on newly calculated mass of organic matter and bulk densities (Deverel and Leighton 2010).

To estimate the consolidation of subsurface deposits, it was assumed that compaction processes are similar to dewatering and irreversible consolidation of a vertical soil column as described by Terzaghi (1925). The use of Terzaghi’s effective stress principle is generally restricted by assumptions of Newtonian behavior of the liquid phase. Water in organic soil does not strictly follow Newtonian mechanical principles, especially during large changes in stress; however, it was assumed that for a small increment of stress change, dewatering would generally follow Newtonian behavior. SUBCALC simulates this process using a linear equation relating compaction to the change in hydraulic head based on data from the Twitchell Island extensometer (Kerr et al. 2003). Effects of varying depth to groundwater were accounted for using the relation of subsidence rates to depth to groundwater described in Stephens et al. (1984). Substantial detail is provided for the original model in Deverel and Leighton (2010, see their Appendix B in their ‘Supplemental materials’ section).

#### Recent model updates

In light of recently available data for land-surface elevation changes and greenhouse-gas emissions from drained Delta organic soils, SUBCALC was modified and re-calibrated for site specific data (Table 1). Specifically, data in Table 1 and information presented in Davidson et al. (2012) were used to estimate *V*_max_ for Eq. 2 as follows.

**Table 1:** Observed versus SUBCALC-simulated subsidence and carbon flux rates

Site (source)	Subsidence^a^ (cm yr^–1^)		Carbon Flux^b^ (g C cm^–2^ yr^–1^)		Soil organic matter content (%)	Average annual depth to groundwater (cm)	Average annual soil temperature (°C)	*V*_max_ (g C cm^–2^ yr^–1^)	*K*_m_
	Observed	Simulated	Observed	Simulated					
Twitchell (this study and Knox et al. 2015)	0.830	0.802	0.057	0.0607	39.7	85	16.4	0.182	0.076
Sherman (this study)	0.515	0.330	–	–	16.9	55	16.0	0.174	*0.128*
Sherman (Hatala et al. 2012)	–	–	0.024	0.023	22.5	50	16.0	0.174	0.115
Staten (Pellerin et al. 2013)	–	–	0.066	0.056	34.0	100	15.0	0.157	0.089
Orwood (Deverel and Rojstaczer 1996)	0.800	0.858	–	–	24.4	139	14.9	0.155	*0.111*
Jersey (Deverel and Rojstaczer 1996)	0.680	0.648	–	–	20.0	108	15.4	0.164	*0.121*

Site-specific *K*_*m*_ values were used to determine a regression equation for model input km as a function of soil organic matter content. *Non-italicized K*_*m*_ *values* are from sites where carbon flux was measured, so *K*_*m*_ was calculated directly (see Eqs. 1 and 2). *Italicized K*_*m*_ *values* are from sites where carbon flux was not measured. These were calibrated to field subsidence data through manual trial-and-error SUBCALC simulations.

^a^For subsidence (observed and simulated), the root mean square error (RMSE) is 0.0991

^b^For carbon flux (observed and simulated), the RMSE value is 0.00605

2 $$ {V}_{\max_{\mathrm{x}}}={a}_{\mathrm{x}}\times {e}^{-\mathrm{E}{\mathrm{a}}_{\mathrm{x}}/RT} $$

Where *a*_x_ is the pre-exponential factor, Ea_x_ is the activation energy for the soil organic carbon oxidation reaction, *T* is soil temperature and *R* is the universal gas constant. Values for *a*_x_ and Ea_x_ were initially extracted from Davidson et al. (2012). Site recorded soil temperatures shown in Table 1 were used.

Using the fraction organic carbon values and calculated *V*_max_ values, *K*_m_ (Eq. 1) was used as a calibration term to match carbon fluxes and subsidence rates in Table 1. A linear relationship between calculated/calibrated *K*_m_ values and soil organic matter content arose. The regression equation was used to estimate *K*_m_ from soil organic matter content values for estimating Delta-wide subsidence rates.

### Estimation of current subsidence rates

The primary spatially variable inputs for SUBCALC are depth to groundwater, soil temperature and soil organic matter content. Michaelis–Menton inputs are calculated from soil temperature and soil organic matter content. The depth of the organic soil where oxidation is simulated to occur is determined by the depth to groundwater and the oxidation rate is governed by depth-to-groundwater/carbon loss-subsidence relations described in Stephens et al. (1984) and Couwenberg and Hooijer (2013). The depth to groundwater was estimated from soil surveys described in the following.

#### Depth to groundwater

Depth to groundwater on Delta subsided islands is controlled primarily by networks of drainage ditches that feed to island drainage pumping stations that in turn continuously discharge drainage water to Delta channels. Drainage ditches collect water that seeps from adjacent channels and deep percolation of applied irrigation water. There are few depth to groundwater measurements in Delta organic soils and, in general, groundwater levels have been maintained at about 0.8–1.2 m below land surface as the result of drainage system operation. Based on the first author’s experience in working in the organic soils throughout the Delta since the early 1980s, depth to groundwater has not changed substantially over time in most places. Also, data presented in Deverel et al. (2015) indicate lack of change in Delta groundwater levels since the late 1980s. To estimate depth to groundwater throughout the Delta for input to the SUBCALC model, information was obtained for each soil type from the soil surveys for Sacramento, San Joaquin, Solano, Yolo, and Contra Costa counties. The soil surveys have an average, or range, of depth to water value for each soil type. Depth to groundwater values were incorporated into a GIS file used to generate a map of estimated depth to groundwater (USDA Soil Survey Staff, Natural Resources Conservation Service 2006, 2007; Welch 1977; McElhiney 1992; Tugel 1993).

#### Soil organic matter content and bulk density

**Fig. 3:**
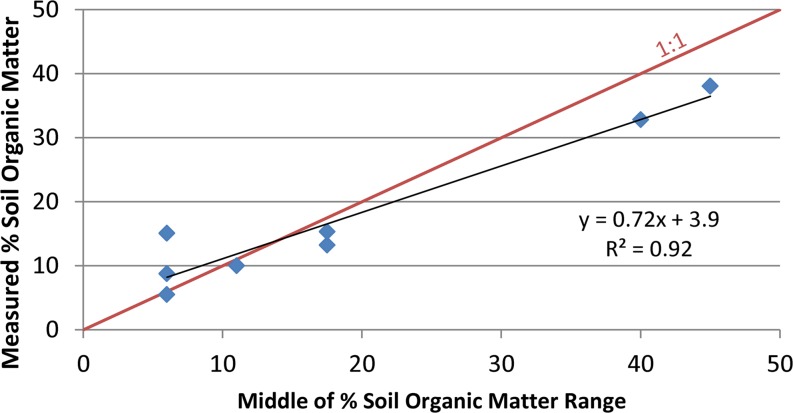
Scatter plot showing relation of average measured soil organic matter content values to middle of range of reported values in the soil surveys

**Fig. 4:**
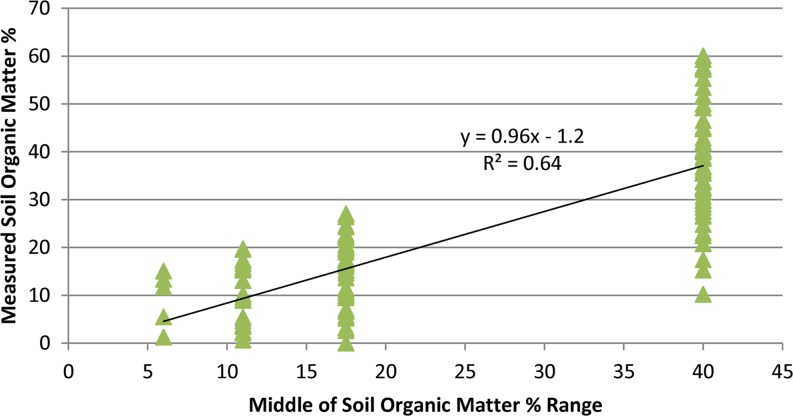
Scatter plot showing relation of measured soil organic matter content values to middle of range of reported values in the soil surveys

**Fig. 5:**
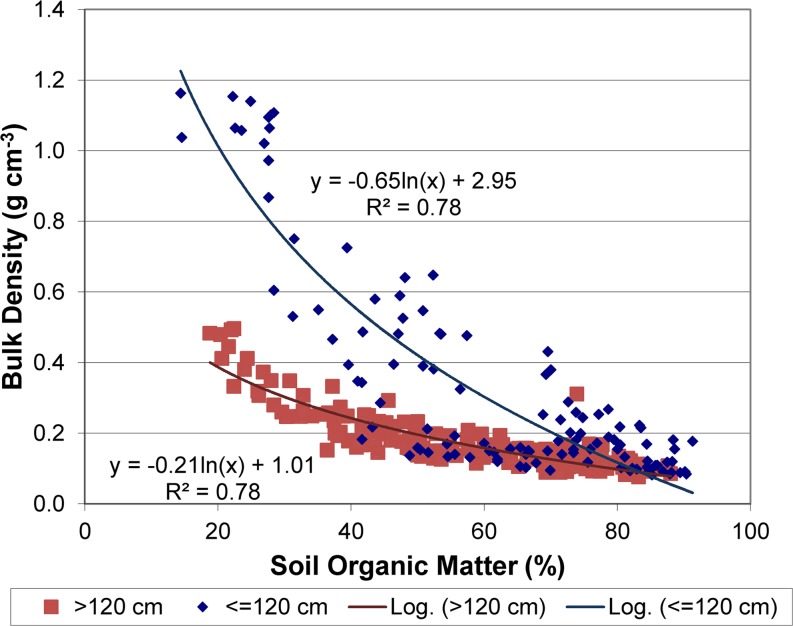
Scatter plot showing the relation of soil bulk density and soil organic matter content on agricultural islands reported in Drexler et al. (2009b) for samples collected above and below 120 cm

Soil organic matter content percentages provided in soil surveys were modified with the results for recently collected soil samples on Twitchell, Staten, Bacon and Sherman islands. Due to oxidation of soil organic matter since collection of data for the soil surveys, available data indicate that present-day soil organic matter content is likely equal to or lower than the mid-range values used to map soil organic matter content in Deverel and Leighton (2010). Soil organic matter determinations on Twitchell and Staten islands during 2012 through 2014 and on Bacon and Sherman islands in 2006 were compared with values reported in the soil surveys and a regression relation was used to estimate present-day values (Figs. 3 and 4). Data presented in Drexler et al. (2009b) for soil percent organic matter and bulk density (Fig. 5) were used to develop a regression relation for estimating initial soil bulk density in SUBCALC.

#### Soil temperature

Spatially referenced monthly near-surface air temperature data were obtained from sources described by Maurer et al. (2002). Data included daily minimum and maximum temperature observations at National Oceanic and Atmospheric Administration (NOAA) Cooperative Observer (co-op) stations. Co-op stations occur at a density of approximately one per 700 km^2^. For the Maurer dataset, these observations were spatially interpolated into a 1/8 ° (degrees latitude-longitude) square grid and averaged monthly.

For each mapped grid cell overlaying Delta organic soils, an annual average temperature for the period 2007–2010 was calculated from all monthly averages. This period was chosen because the 1950–1999 data exhibit an upward trend. It was assumed that the 2007–2010 data adequately represent present-day Delta temperatures. Each soil feature in the GIS shapefile from this study had a temperature assigned to it from its corresponding grid cell. Air and soil temperature data from Knox et al. (2015) and Hatala et al. (2012) were compared to adjust the air temperatures for SUBCALC model input. On average, soil temperature was 1.2 °C greater than air temperature. This difference was used to convert air to soil temperature for input into Arrhenius function calculations within SUBCALC (Eq. 2).

#### Mapping of present-day subsidence

Spatially variable present-day subsidence rates were estimated using the recalibrated SUBCALC model and ArcGIS Spatial Analyst. It was assumed that oxidation and consolidation are the only present-day causes of subsidence. It was also assumed that there will be zero subsidence in rice-growing areas and permanently flooded wetlands, and the subsidence rate is zero where the soil organic matter content is less than or equal to 2 %.

## Results

### Recently measured and estimated subsidence rates

#### Extensometer and leveling data

**Fig. 6:**
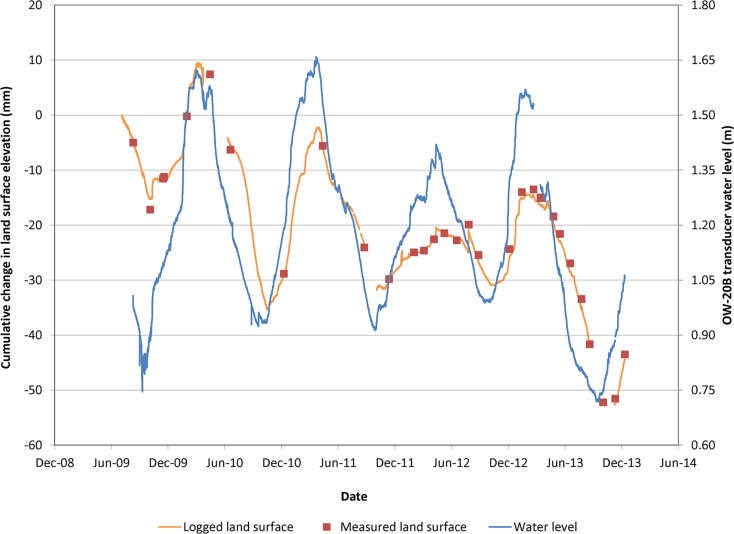
Time-series plot of land-surface elevation changes and groundwater level, Twitchell Island extensometer in cornfield

Twitchell Island cornfield extensometer and observation-well data (Fig. 6) illustrate seasonal variations in land-surface elevations associated with groundwater-level fluctuations from 2009 through 2013. Groundwater levels rose during fall primarily due to decreased crop evapotranspiration and winter precipitation recharge. Groundwater levels decreased in the spring with diminishing rain and increasing evapotranspiration. Using land-surface elevation measurements at times of equal groundwater levels in October 2009 and December 2013, an average inelastic subsidence rate of 0.83 cm yr^–1^ was estimated. The soil organic matter content was 39.7 % (Table 1). For the seven locations where elevations were determined at observation wells in 2001 and 2012, subsidence rates ranged from 0.11 to 1.94 cm yr^–1^ in agricultural fields where estimated soil organic matter content ranged from 6 to 20 %. Generally consistent with extensometer data, the average of all seven measurements during 2001–2012 was 0.7 cm yr^–1^.

**Fig. 7:**
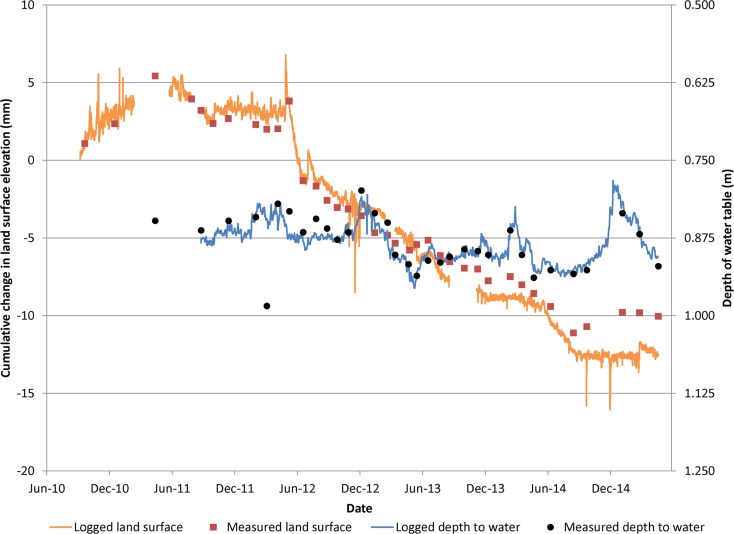
Time-series plot of land-surface elevation changes and groundwater level, Sherman Island

The Sherman Island extensometer was located next to a drainage pumping station which maintained groundwater levels relatively constant with time since April 2011 (Fig. 7). The inelastic subsidence rate during April 2011 to April 2015 was 0.52 cm yr^–1^. Soil organic matter content was 16.9 % in 2015 (Table 1).

#### Twitchell rice leveling and extensometer data

**Fig. 8:**
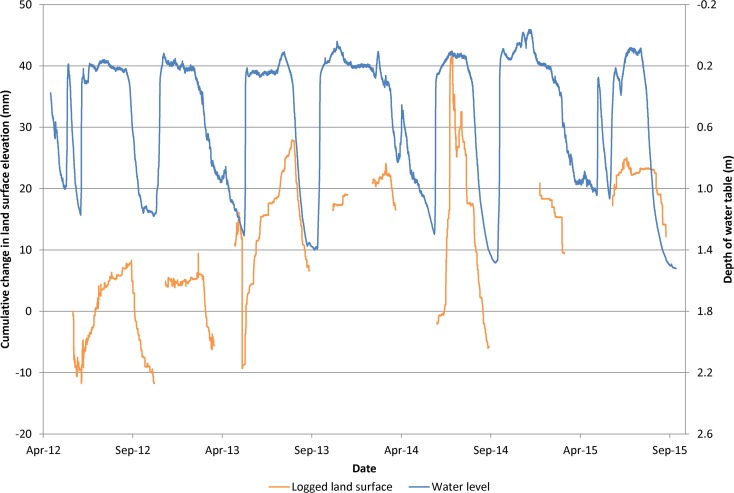
Time-series plot of land-surface elevation and depth to groundwater at Twitchell Island rice extensometer

The results of surveying in Twitchell Island rice fields indicated average elevation changes ranging from −1.7 to 2.1 cm yr^–1^ from 2009–2013. The average elevation-change rate for all seven locations in the rice fields was 0.05 cm yr^–1^. Spatial variations in rates are primarily due to land disturbance resulting from agricultural activities which include disking for weed control and preparation for planting, harvest and subsequent incorporation of plant residues. During 2012–2015, extensometer data from the Twitchell Island experimental rice field exhibits elastic and inelastic land-surface elevations and changes associated with seasonal cycles in groundwater level changes (Fig. 8). Flooded conditions were maintained in the rice fields during the growing season (late spring through late summer) and during the winter. Fields were drained before seeding in the spring and harvest in late summer/early fall. Six periods of flooding and draining are evident from 2012–2015 (Fig. 8).

The 2012 data illustrate key events in the rice cultivation cycle. Land surface elevation initially followed the decline in the groundwater levels as the field was dewatered prior to planting. After planting and through the growing season, groundwater levels were about 20 cm below land surface at the observation well. During that period, land-surface elevations steadily increased. At the end of the growing season, the field was dewatered for harvest in September and groundwater levels and land-surface elevations decreased. The SET was moved off the field for harvesting and cultivation. When the SET was returned later to the exact same location in the field on 6 December 2012, land surface had risen by 17 mm, relative to the previous measurement on 11 November 2012 due to increased groundwater levels resultant from flooding and land disturbance. The water table was maintained through the winter at roughly the same level as during the growing season. The field was dewatered for preparation for planting beginning in late February at which point land-surface elevations declined to about the same level measured in November 2012 as shown by the manual measurement in March 2012. Similar oscillations were observed during 2013–2015.

**Fig. 9:**
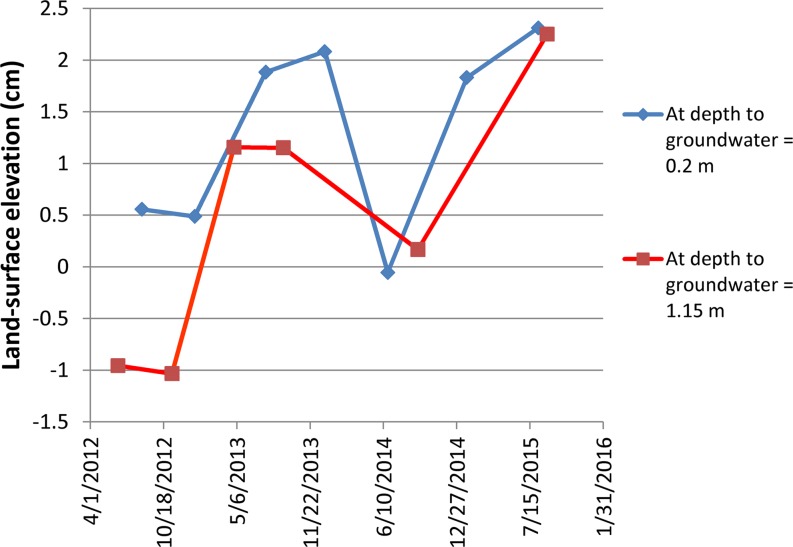
Time-series plot of land-surface elevation for equal depth to groundwater

Net changes in land-surface elevations were estimated from the data shown in Fig. 8 by comparing annual land-surface elevation measurements at times when groundwater levels were equal. Land-surface elevations measured when the groundwater levels were shallowest (0.2 m) and deepest (1.15 m) indicated an overall average net accretion of about 0.8 cm yr^–1^ (Fig. 9). Specifically, the average accretion rates for the shallow (0.2 m) and deep groundwater depths (1.15 m) were 0.47 and 1.2 cm yr^–1^, respectively. The average accretion rate for all measurements was 0.84 cm yr^–1^.

### Estimation of Delta-wide subsidence

#### Distribution of soil organic matter

**Fig. 10:**
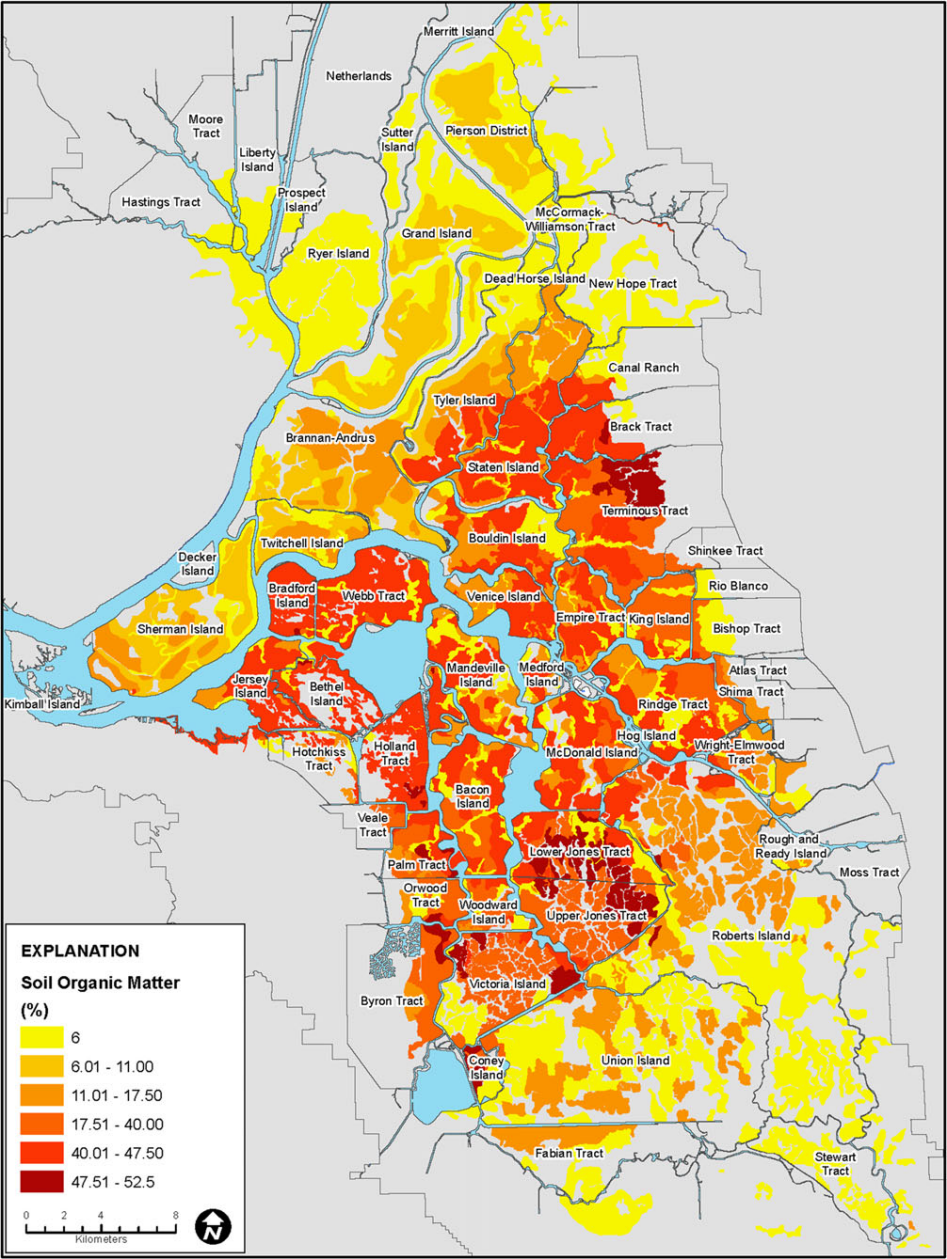
Map showing distribution of soil organic matter percentage, Sacramento-San Joaquin Delta

Figure 3 indicates that relative to values reported in the soil surveys, average soil organic matter has decreased over time and that average values are likely about 72 % of the reported values where there is over 20 % soil organic matter content; however, Fig. 4 indicates that overall, the medians of recently measured values were slightly lower than the mid-range of soil-survey reported values as indicated by the slope of 0.96. The average soil organic-matter percentage values in Fig. 10 were based on the data from soil surveys.

Mapped soil organic matter content varied from less than 6 % to over 52 % throughout the Delta (Fig. 10). The distribution of soil organic matter reflects geomorphologic and subsidence history. Highly organic mineral surface soils generally predominate in the western and northern Delta and organic soils are prevalent in the central, eastern and south-central Delta. The lowest organic matter content soils generally prevail in areas drained prior to 1880 near the Sacramento River. In contrast, central and eastern Delta islands, where higher organic-matter soils dominate, were reclaimed during the late 19th century or early 20th century. Prior to reclamation, islands near the Sacramento River (e.g. Sherman Island) were subject to greater fluvial deposition relative to the more quiescent environment in the central and eastern Delta.

#### Distribution of soil temperature

There is little spatial variation in near-surface annual air temperature. Average annual temperatures ranged from 16.25 to 17.25 °C for the entire Delta.

#### Distribution of depth to groundwater

There are generally small variations in depth to groundwater in the Delta due to the influence of drainage systems. Based on information in the soil surveys, average depth to groundwater ranged from 0.8 to 1.2 m for most of the organic-soil area. Exceptions included the western Delta (notably Sherman and Jersey islands where pasture is the predominant land use). In these areas, depth to groundwater levels varied from 0 to 0.8 m.

#### Estimated subsidence rates

The SUBCALC model was re-calibrated for data for land-surface elevation change and greenhouse gas emissions (Table 1) collected throughout the Delta (Fig. 1). Average depth to groundwater varied from 0.50 to 1.39 m and soil organic matter content varied from 14.9 to 39.7 %. There was a small average annual temperature variation among the sites (14.9–16.4 °C) and therefore small variability in *V*_max_. The values of *K*_m_ varied inversely with soil organic matter content. The root-mean square error (RMSE) was calculated as a goodness of fit parameter. For subsidence where depth to groundwater, soil temperature and soil-organic matter content are known, RMSE values indicated that model predictions were accurate within ±0.10 cm yr^–1^, whereas carbon fluxes were accurately predicted within ±0.006 g C cm^–2^ yr^–1^.

**Fig. 11:**
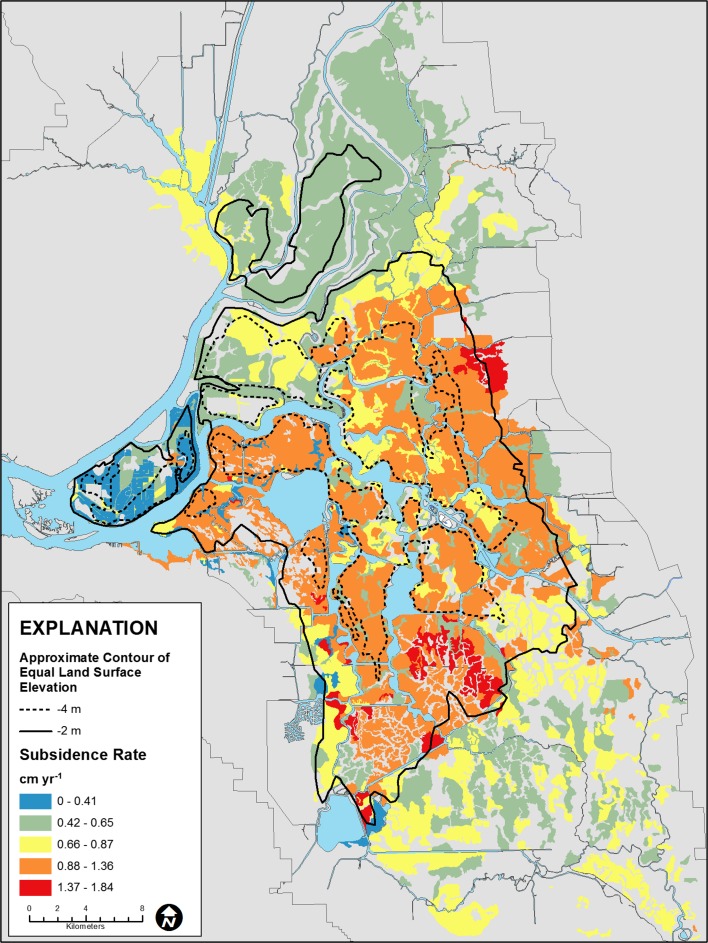
Map showing distribution of present-day modeled subsidence rates

Estimated present-day subsidence rates, which varied from 0.28 to 1.8 cm yr^–1^ based on inputs for depth to groundwater, average annual soil temperature and soil organic matter content are shown in Fig. 11. The highest rates (over 0.9 cm yr^–1^) correspond to high organic-matter soils in the central Delta where estimated soil organic matter content was over 40 % (Figs. 10 and 11). Rates generally equal to or lower than 0.9 cm yr^–1^ corresponded to the western Delta where soil organic matter content generally ranged from less than 6 to over 17 %. Estimated subsidence was also low or nil in the northern, eastern and southern Delta where organic matter contents were generally less than 15 %. At locations where rice cultivation and wetlands have been implemented (Sherman, Twitchell, Brack, Canal Ranch, Wright Elmwood), zero subsidence was assumed; active subsidence occurs where there is peat at or below elevation –2 m and the highest rates occur below –4 m (Fig. 11).

## Discussion

### Subsidence rates

Measured present-day subsidence rates vary substantially in the Delta and are primarily related to soil organic matter content and secondarily to water- and land-management practices, which determine depth to groundwater. Deverel and Leighton (2010) measured land-surface elevations at 51 locations in 2006 where Weir and colleagues determined elevations in 1978 on Bacon Island (Fig. 1). Subsidence rates ranged from 1.5 to 3.0 cm yr^–1^ where soil organic matter content varied from 15 to over 60 %. On Sherman Island, Deverel and Leighton (2010) resurveyed power-pole foundations in 2006 originally surveyed in 1988 by Rojstaczer et al. (1991) and reported subsidence rates ranging from 0.6 to 2 cm yr^–1^ where soil organic matter content varied from 3 to 20 %.

Figure 11 shows generally lower rates for most of the Delta relative to those reported by Deverel and Leighton (2010). Two key factors explain lower subsidence rates: lower soil organic matter content resultant from ongoing oxidation, and recalibration of SUBCALC with more recent data for subsidence and carbon emissions. For example on Bacon Island (Fig. 1), Deverel and Leighton (2010) reported soil organic matter contents over 60 %. Using the adjusted values described in the ‘Data sources and methods’ section resulted in lower present-day average organic matter content and subsidence rates for the eastern portion of Bacon Island as ranging from 0.9 to 1.4 cm yr^–1^. The average subsidence rate was 1.3 cm yr^–1^. For comparison, Deverel and Leighton (2010) reported an average rate of 2.2 cm yr^–1^ for this area from 1978 to 2006. Within any soil type, soil organic matter content can vary substantially (Fig. 4). The SUBCALC-based subsidence maps presented here therefore provide spatially averaged rates that are representative of areas of similar soil organic matter content at the level of tens to hundreds of hectares.

To better assess SUBCALC-simulated present-day subsidence rates for varying organic matter content, on Bacon Island, present-day rates were simulated at the locations where Deverel and Leighton (2010) reported 1978–2006 subsidence rates ranging from 1.5 to 3.8 cm yr^–1^ and the average rate was 2.2 cm yr^–1^, whereas soil organic matter content ranged from 14 to 60 % and the average soil organic matter content was 39 %. SUBCALC present-day rates ranged from 0.7 to 2.0 cm yr^–1^ for the same range of organic matter content percentages, with the average rate being 1.2 cm yr^–1^.

A similar comparison was made between SUBCALC-simulated present-day subsidence rates and Sherman Island 1988–2006 subsidence rates, also reported in Deverel and Leighton (2010). Among Sherman Island measurement points, subsidence rates ranged from 0.6 to 2.0 cm yr^–1^ and the average rate was 1.2 cm yr^–1^, while soil organic matter content ranged from 3 to 20 % with an average of 10 %. SUBCALC present-day rates ranged from 0.3 to 0.6 cm yr^–1^ for the same range of organic matter content percentages, and the average rate was 0.4 cm yr^–1^. These comparisons illustrated the likely variation within a central (Bacon) and western (Sherman) Delta island and indicates that current subsidence rates are lower than rates in previous decades.

Priyanka Sharma and colleagues (P. Sharma, Jet Propulsion Laboratory, California Institute of Technology, personal communication, 2015) used UAVSAR data to estimate subsidence rates from 2009 to 2014 over all of Sherman Island. Reported rates ranged from 0 to 5 cm yr^–1^ and averaged 1.5 cm yr^–1^. By comparison, SUBCALC-simulated subsidence rates ranged from 0.3 to 1.1 cm yr^–1^ and averaged 0.5 cm yr^–1^ at Sherman Island. Within the Sharma study, the UAVSAR method was found to have over-predicted the subsidence rate (0.7 cm yr^–1^) at the location of an extensometer, which measured a rate of 0.4 cm yr^–1^ over the same period. The reported uncertainty in the UAVSAR estimates is about ≤ 0.2 cm yr^–1^.

### Rice as subsidence-mitigation land use

Direct (elevation-change measurements) and indirect estimates (micrometeorological and organic-matter mineralization) of subsidence and accretion are generally consistent in preliminarily evaluations indicating that rice stops or greatly reduces subsidence by providing above- and below-ground plant residue which is incorporated into the soil and by reducing the rate of peat oxidation under saturated conditions. Data derived from leveling surveys and the extensometer demonstrate substantial temporal and spatial variability in land-surface elevation changes and indicate a small net accretion rate. These data illustrate the difficulty in estimating short-term land-surface elevation changes in this and other systems where there is land disturbance and elastic shrinking/swelling changes associated with short-term and seasonal groundwater elevation and soil moisture content changes (e.g. Zanello et al. 2011). Similar difficulties were noted with data collected by Weir (1950) in Deverel and Leighton (2010). Longer-term, high-quality land-surface elevation measurements for rice will provide more definitive answers.

Reported subsidence estimates in the Twitchell Island rice fields from indirect methods indicate small rates of subsidence. Based on the eddy-covariance determination of carbon loss, Hatala et al. (2012) estimated subsidence in the Twitchell Island rice field at 0.1 to 0.14 cm yr^–1^ during 2 years. During a 1-year study, Kirk et al. (2015) estimated soil organic matter-nitrogen mineralization rates at four locations in the Twitchell Island rice field and used these with soil carbon:nitrogen ratios to estimate subsidence rates ranging from 0.07 to 0.11 cm yr^–1^, in close agreement with results reported by Hatala et al. There is uncertainty in these indirect estimates.

In estimating subsidence rates using nitrogen mineralization from peat, Kirk et al. (2015) determined the annual nitrogen budget and by accounting for fertilizer application and plant uptake, calculated the annual mineralized nitrogen as a source to plant nitrogen uptake. Nitrogen from groundwater was estimated in situ using groundwater and soil-water samples in mesocosms. Kirk et al. (2015) assumed that groundwater nitrogen contributions resulted from mineralization during the year of investigation. Given low hydraulic conductivity and high porosity of the organic soils, groundwater nitrogen likely resulted from mineralization during previous years. Additionally, Kirk et al. (2015) applied an f_min_ factor of 0.67 based on Deverel and Leighton (2010), a factor that is not applicable because any subsidence in rice fields results from oxidation of the organic soil. In Deverel and Leighton (2010), the value of 0.67 was the estimated fraction of organic soil oxidation contributing to subsidence originally reported in Deverel and Rojstaczer (1996). The remaining fraction was attributed to consolidation due to deepening of drainage ditches. Because the rice field drainage ditches have not been altered, the sole cause of subsidence would be oxidation of soil organic matter. Removing the groundwater contribution and f_min_ factor from the Kirk et al. (2015) calculations resulted in a subsidence rate of about 0.02 cm yr^1^; furthermore, Kirk et al. (2015) used a plant nitrogen uptake efficiency of 50 % derived from the literature for mineral soils and fertilizer and stated that the values could range as high at 70 %. Using the 70 % value in Kirk et al.’s (2015) calculation and removing the groundwater contribution and the f_min_ factor denominator resulted in accretion (0.001 cm yr^1^) to a small amount of subsidence (0.01 cm yr^1^).

Hatala et al. (2012) estimated the net carbon balance (carbon dioxide sequestered – methane emitted + planted seed – harvested grain) and used data for soil bulk density and soil carbon to estimate subsidence rates in rice during 2009–2011. The range of carbon balance values presented was used here to estimate subsidence values as low as 0.07 cm yr^1^. Using data presented in Knox et al. (2015) for 2013, subsidence rates ranging from 0.02 to 0.13 cm yr^1^ were estimated. The lower range of estimates of subsidence and accretion based on the data published in Hatala et al. (2012), Knox et al. (2015) and Kirk et al. (2015), are more consistent with measured land-surface elevation changes which indicate a small rate of overall accretion. Qualitatively and in light of uncertainty, the preponderance of evidence summarized here preliminarily indicates that rice cultivation greatly reduces subsidence or may slightly reverse the effects of subsidence. Longer-term data will provide improved quantification of the long-term subsidence mitigation benefit due to rice cultivation. Knox and colleagues (S. Knox, University of California-Berkeley, personal communication, 2016) presented results of 6 years of eddy covariance measurements of CO_2_ and CH_4_ fluxes in the Twitchell Island rice field. These data show heretofore unreported substantial annual variability in photosynthesis and methane fluxes driven primarily by variability in soil temperatures and resulted in substantial variability in soil carbon budgets.

### Mitigation

The primary Delta subsidence mitigation tools are rice cultivation and permanently flooded wetlands. As demonstrated here and elsewhere (e.g. Miller et al. 2008; Deverel et al. 2014), both of these land use practices stop, greatly reduce or reverse the effects of subsidence. The work described here and in Deverel et al. (2014) provide guidance for implementation of these land-use changes. Areas below elevations of –2 m are candidate areas for implementation because there is active subsidence occurring (Fig. 11). Moreover and consistently, Deverel et al. (2015) demonstrated that artesian conditions prevail below –2 m and 81 % of wet, non- or marginally farmable areas were at or below –2 m. Implementation of rice and wetlands in these areas will prevent or reduce subsidence and associated consequences.

## Summary and conclusions

Subsidence due primarily to oxidation of soil organic matter in the Sacramento-San Joaquin Delta affects sustainability of California’s water supply system and local agriculture. By reducing the landmass and resistance to hydraulic pressure from adjacent channels, subsidence has contributed to levee failure and island inundation which potentially affects water for use by over 25 million Californians and irrigation of millions of hectares of agricultural land. Since the mid-nineteenth century, oxidation has resulted in up to 8 m of subsidence. Subsidence rates have declined with time due to the disappearance of about 2.5 billion m^3^ of organic soil and consequent decreases in soil organic matter content and changing management practices. Present-day Delta subsidence rates have not heretofore been extensively recorded or estimated.

Land-surface elevation data were collected to assess present-day subsidence rates and preliminarily evaluate rice as a land use for subsidence mitigation. To depict Delta-wide present-day rates of subsidence, the previously developed and reported SUBCALC model was revisited and calibrated using recent subsidence rates and carbon flux data. The primary inputs to the SUBCALC model include depth to groundwater, soil organic matter content and soil temperatures which were spatially estimated using multiple data sources. These inputs were used to map estimated subsidence rates. Land-surface elevation change data was collected and evaluated relative to indirect estimates of subsidence and accretion using carbon and nitrogen flux data for rice.

Extensometer data in a cornfield on Twitchell Island demonstrate seasonal variations in land-surface elevations associated with groundwater-level fluctuations from 2009 through 2013 and an inelastic subsidence rate of 0.83 cm yr^–1^. Leveling data resulted in a similar estimated subsidence rate of 0.7 cm yr^–1^ from 2000–2012 on Twitchell Island. The Sherman Island extensometer data indicated a rate of 0.52 cm yr^–1^ where there was lower soil organic matter content. Calibration of the SUBCALC model indicated accuracy of ±0.10 cm yr^–1^ where depth to groundwater, soil organic matter content and temperature are known, while regional estimates of subsidence based on spatial variations in estimated soil organic matter content, depth to groundwater and soil temperature range from less than 0.3 to over 1.8 cm yr^–1^. The primary uncertainty is the distribution of soil organic matter content which results in spatial averaging in the mapping of subsidence rates at the level of tens to hundreds of hectares.

Analysis of leveling and extensometer data in the Twitchell Island rice field resulted in an estimated accretion rate of 0.02 to 0.8 cm yr^–1^. Indirect estimates based on measurements of carbon fluxes and nitrogen mineralization resulted in estimates of low subsidence rates to low accretion rates. The preponderance of evidence presented here preliminarily demonstrates that rice will stop or greatly reduce subsidence for most of the Delta. Areas below elevations of –2 m are candidate areas for implementation because there is active subsidence occurring at rates greater than 0.4 cm yr^–1^.
